# Machine Learning for Biomedical Applications

**DOI:** 10.3390/bioengineering11080790

**Published:** 2024-08-05

**Authors:** Giuseppe Cesarelli, Alfonso Maria Ponsiglione, Mario Sansone, Francesco Amato, Leandro Donisi, Carlo Ricciardi

**Affiliations:** 1 Department of Electrical Engineering and Information Technology, University of Naples Federico II, Via Claudio 21, 80125 Naples, Italy; alfonsomaria.ponsiglione@unina.it (A.M.P.); msansone@unina.it (M.S.); framato@unina.it (F.A.); carloricciardi.93@gmail.com (C.R.); 2 Department of Advanced Medical and Surgical Sciences, University of Campania Luigi Vanvitelli, Via De Crecchio 7, 80138 Naples, Italy; lean.donisi@gmail.com

Machine learning (ML) is a field of artificial intelligence that uses algorithms capable of extracting knowledge directly from data that could support decisions in multiple fields of engineering. Particularly in the last few years, calculating capacity has progressed and done so at the same speed as ML algorithms, a situation which potentiates the outperformance/replacement of several classical methods. This paradigm has also been confirmed in the context of the biomedical sector. For instance, previous research has documented the introduction of ML algorithms in studies aimed at disease diagnosis and monitoring based on biomedical signals and/or data [1,2,3,4,5,6] or on biomedical images [7,8,9,10]. Considering the great interest in these topics, this Special Issue (SI) sought high-quality contributions that described novel applications for ML strategies in the context of the biomedical field.

Table 1 shows the 21 contributions proposed to this SI so as to provide an overview of the topics addressed by the research groups that selected this SI as the best-suited platform in which to publish their studies.

Medical/biomedical imaging technology was mainly used by research groups that have proposed a contribution to this SI. In this last field, ML has gradually interested the authorized personnel since it has been demonstrated to potentially better support clinicians’ decisions with respect to the use of the imaging techniques alone. These aspects have been investigated by two reviews/surveys proposed to this SI. Saleh et al. focused on ophthalmic imaging modalities to diagnose, detect, and stage retinal diseases (in particular, the Authors investigated age-related macular degeneration and diabetic retinopathy), which currently are mainly fundus imaging, optical coherence tomography, and optical coherence tomography angiography [11]. Therefore, the Authors collected studies that described applications where images were then fed to artificial intelligence (AI)-based methods, such as both traditional ML algorithms and deep learning (DL) pipelines; albeit, the Authors found that both strategies effectively helped to diagnose, detect, and stage retinal diseases. Saleh et al. conclude with their belief that only DL will eventually become state-of-the-art being better devised for automated image interpretation goals (even by mobile applications). AI methods have also proven effective in improving the performance of non-invasive diagnostic approaches, such as conventional ultrasonography, used for detecting and quantifying non-alcoholic fatty liver disease [12]. Like the former survey, both ML and DL methodologies have been reported; however, again, DL strategies, e.g., artificial neural networks (ANN), to the best of the Authors’ knowledge, will be a significant advancement from classical ML for their capability of using data to learn high-level features (even if, the comparison among computational complexity/power and classification accuracy should be considered another important aspect for choosing DL methods with respect to ML methods). Considering again imaging for liver healthcare, Popescu et al. proposed an intelligent decision system for segmenting liver and hepatic tumors by integrating four efficient neural networks (ResNet152, ResNeXt101, DenseNet201, and InceptionV3) [13]. The proposed system effectively overperformed the single networks from which it is composed, although the Authors claimed that the pipeline still needs adjustments and parameter fine-tuning to be on par with state-of-the-art methods, such as improved UNets. Similarly, Abu Haeyeh et al. presented a multiscale weakly supervised DL approach to classify renal histopathology images, in particular, to distinguish between benign tissue and malignant renal cell carcinoma tumors and identify the tumor subtypes to support medical therapy management [14], achieving scores greater than 90%. Furthermore, addressing a related issue, El-Melegy et al. proposed a kidney image segmentation method based on fuzzy c-means and statistical shape models, a method which outperformed several state-of-the-art-level set methods with an average improvement of 0.7 in terms of the 95-percentile Hausdorff distance [15]. Finally, Ukwuoma et al. [16] and Qi et al. [17] found similar results when analyzing endoscopic images for ulcerative colitis and chest X-ray images for lung diseases.

Other contributions of ML similar to medical/biomedical imaging proposed to this SI are those of ElNakieb & Ali [18], Pepetuini et al. [19], and Kim et al. [20]. The former paper [18] describes a pipeline which, starting from a dataset of brain images—acquired by functional magnetic resonance imaging (fMRI)—of subjects affected by autism spectrum disorder (ASD), enabled the preprocessing of images (i) for the analysis of connectomes, (ii) performance of brain parcellation, (iii) feature representation, (iv) feature selection, and (v) ML classification. In the context of the aforementioned ML classification, ML was used both for feature selection and then for autistic brain classification, following which the algorithms achieved a balanced accuracy of 98.8% using five-fold cross-validation. Otherwise, the other two papers faced different issues using information from video-measurements technologies. For instance, Perpetuini et al. [19] provided evidence suggesting the combination of infrared thermography and ML could automatically detect peripheral circulation impairments in Alzheimer’s disease patients compared to healthy controls (HCs). In particular, after recording facial videos of both patients and selecting a specific region of interest for the accurate detection of peripheral circulation impairments, data were analyzed to compute and select specific features and to set up a supervised ML classification. Decision tree demonstrated the most accurate (82%) algorithm among the various tested, an aspect which indicates that alterations in microvascular patterns in Alzheimer’s disease patients could be detected in the early stages of the pathology using the presented strategy. Similarly, Kim et al. [20] used a smartphone camera to acquire face images to be, in turn, analyzed to study the photoplethysmography (PPG) signal by an improved (respect to the current state-of-the-art) DL pipeline based on a time-series feature analysis that uses axial projection of the images. The improved pipeline provided evidence of a better evaluation of PPG in a short time on two datasets.

In this SI, ML has also been demonstrated to improve pipelines in the field of biosignal processing. Considering the PPG signal again, Samimi & Dajani [21] presented a method to non-invasively and cufflessly estimate blood pressure, considering both dynamic changes of the pulse waveform and pulse transit time. The parameters and features extracted from these data (pertinent to 30 patients) were fed to an ANN, which was performant and accurate, indicating the feasibility of the proposed pipeline and suggesting potential further synergies with other methods [21]. Considering useful signals to detect potential cardiovascular diseases, Rabbani & Khan proposed a contrastive self-supervised learning approach to support electrocardiography (ECG)-based stress assessments [22]. In particular, firstly, a neural network model was trained to learn ECG data (not considering ground truth labels), information which, in the subsequent supervised task, was considered crucial to set network weights used for initialization in the training stage of the model. Overall, the pipeline overperformed other already published models fed by the same two datasets [22]. Human movement was another biomedical issue faced by several Authors who selected the SI. For instance, contrary to [18], Pradhan et al. [23] studied the impaired movement patterns of ASD patients, setting up a multisegment foot mechanical model capable of extracting kinematic features, which, opportunely combined, proved evidence to classify autism gait patterns. In particular, support vector machine models (which proved more performant than other models) used in this work demonstrated an improved accuracy when fed with multisegment foot-related features with respect to the traditional single-segment foot-related features [23]. Contrarily, Asfour et al. [24] investigated specific aspects of gesture recognition using surface electromyography and ML. Starting from the evidence that offline gesture recognition and real-time applications experience an accuracy bias of about 10%, the Authors set up a feature–classifier pairing compatibility study, following which they were able to provide a guide—based on the analysis of more than 40,000 features and classifiers pairs—to properly choose features and classifiers which should support the reduction of the initial bias [24]. The recent health emergency has also opened up new opportunities in the biomedical signal-processing field. Among these, Abdeltawab et al. [25] proposed one pertinent to respiratory support for hospitalized inpatients. The Authors retrospectively collected anamnestic and clinical data of about 3500 patients and, after several data preparation and feature selection operations, set up a three-class supervised ML analysis using five algorithms. Overall, the algorithms proved capable of distinguishing the level of needed respiratory support (i.e., minimal, non-invasive, invasive), and the XGBoost algorithm reported the best classification accuracy [25].

Furthermore, ML has proved to support investigations also in other biomedical specialties, which founded their investigations on either computational models, bioinformatic tools, or statistical analysis. For instance, considering the first technique, Leong et al. [26] proposed a surrogate model based on ML and finite elements modeling to provide abdomen mechanics predictions in a shorter time compared to traditional computational models. The surrogate model (based on ANN) demonstrated the capability of effectively predicting abdomen palpation stress distribution and forces in a few fractions of a second (instead of more than an hour) with an accuracy (with respect to the simulations) of more than 90%. Similarly, Padhee et al. [27] proposed a combined pipeline based on angiography images and ML to predict hemodynamic characteristics of arterial models. In this case, the objective was to estimate the velocity field of a simile contrast perfusion media in blood, starting from angiography images of the phenomena, compared with simulation results. The findings were very promising since the alternative model greatly lowered the error rate of the computational models [27]. Regarding bioinformatic applications, Thrun et al. and Bakare et al. proposed new contributions for the detection of acute promyelocytic leukemia [28] and pneumonia [29]. In the former paper, the Authors set up a sequential, combined ML unsupervised (Bayesian) task and statistical analysis (ABC analysis); this strategy proved able to reveal which specific cluster of differentiation genes allowed a more straightforward distinction between acute promyelocytic leukemia from non-promyelocytic acute myeloid leukemia [28] since current large-scale sequences and gene-expression studies demonstrated a limited practical clinical value. Similarly, Bakare et al. started their investigation on antimicrobial peptides for pneumonia detection using a tool to identify their binding to pneumonia pathogens receptors [29]. Finally, two studies—submitted by researchers of Southern Italy academic and clinical institutions—proposed to model the length of stay (LOS) in hospital for patients with femur fracture [30] and with potential stroke (which can be limited by endarterectomy surgery) [31] by using ML strategies, since inpatients can experience post-surgery complications and, consequently, prolonged LOS. In both cases, a supervised ML task was set up to predict LOS using as input several anamnestic and clinical parameters. In both cases, the algorithm showed promising classification scores (around 80% accuracy for the best algorithms), which, consequently, indicated these techniques could support investigations aimed at improving the healthcare management of hospitals, reducing LOS (and, in turn, healthcare costs).

In conclusion, this SI has collected a consistent number of contributions in which the Authors proposed the use of ML strategies (for different purposes) on data extracted from medical/biomedical imaging, mainly or differently from biomedical signals to be processed. In addition, minor contributions to this SI were oriented to propose valuable alternatives for traditional computational methods or bioinformatic methods and, finally, show effective applications of ML also to face health management issues. Pointing out that these categories represent only a small portion of the possible applications of ML to biomedical issues, the promising findings described in the literature show that continued research in this field appears fully justified, so that within this journal, a Volume II for the SI has been opened for collecting novel supplementary contributions of significant value. Certainly, these would also aim at promoting improvements in biomedical applications considering the potential information included in health big data and considering novel integration with existing strategies, which could hinder the (inevitable) limitations that ML strategies present when facing biomedical applications [4,32].

## Figures and Tables

**Table 1:** Topics and goals investigated by the papers published in this Special Issue. Abbreviations. AD: Alzheimer’s disease. AMD: age-related macular degeneration. AMPs: antimicrobial peptides. ASD: autism spectrum disorder. DCE-MRI: contrast-enhanced magnetic resonance images. DL: deep learning. DR: diabetic retinopathy. FE: finite element. fMRI: functional magnetic resonance imaging. HCs: healthy controls. IRT: infrared thermography. ML: machine learning. MSF: multisegment foot. PPG: photoplethysmography. PHF3: pyramid hybrid feature fusion framework. rPPG: remote photoplethysmography. sEMG: surface electromyography. US: ultrasound.

Group		Author/s	Title	Goal
Reviews		Saleh et al. [11]	The Role of Medical Image Modalities and AI in the Early Detection, Diagnosis and Grading of Retinal Diseases: A Survey	Investigating imaging modalities for accurate diagnosis, early detection, and staging of both AMD and DR and the role of AI in automating such operations
		Alshagathrh & Househ [12]	Artificial Intelligence for Detecting and Quantifying Fatty Liver in Ultrasound Images: A Systematic Review	Investigating how well various AI methods function and perform on US images to diagnose and quantify non-alcoholic fatty liver disease
Medical/biomedical imaging		Popescu et al. [13]	Decision Support System for Liver Lesion Segmentation Based on Advanced Convolutional Neural Network Architectures	Proposing an intelligent decision system for segmenting liver and hepatic tumors by integrating four efficient neural networks
		Abu Haeyeh et al. [14]	Development and Evaluation of a Novel Deep-Learning-Based Framework for the Classification of Renal Histopathology Images	Proposing a multiscale weakly supervised deep learning approach for RCC subtyping to support medical therapy management
		El-Melegy et al. [15]	Level-Set-Based Kidney Segmentation from DCE-MRI Using Fuzzy Clustering with Population-Based and Subject-Specific Shape Statistics	Presenting a DCE-MRI-based kidney segmentation method based on fuzzy c-means and statistical shape models
		Ukwuoma et al. [16]	Automated Lung-Related Pneumonia and COVID-19 Detection Based on Novel Feature Extraction Framework and Vision Transformer Approaches Using Chest X-ray Images	Constructing a reliable DL model capable of producing high classification accuracy on chest X-ray images for lung diseases
		Qi et al. [17]	PHF3 Technique: A Pyramid Hybrid Feature Fusion Framework for Severity Classification of Ulcerative Colitis Using Endoscopic Images	Proposing the DL-based PHF3 technique as an auxiliary diagnostic tool for clinical UC severity classification
		ElNakieb & Ali [18]	Understanding the Role of Connectivity Dynamics of Resting-State Functional MRI in the Diagnosis of Autism Spectrum Disorder: A Comprehensive Study	Presenting a pipelined framework based on fMRI aimed at an accurate ASD diagnosis (also of the brain regions contributing to the diagnosis decision)
		Perpetuini et al. [19]	Altered Microcirculation in Alzheimer’s Disease Assessed by Machine Learning Applied to Functional Thermal Imaging Data	Investigate peripheral microcirculation impairments in AD patients with respect to age-matched HCs at resting state through IRT and ML approaches
		Kim et al. [20]	A Study of Projection-Based Attentive Spatial–Temporal Map for Remote Photoplethysmography Measurement	Proposing a DL-based method for elaborating rPPG signal
Biomedical signal processing		Samimi & Dajani [21]	Cuffless Blood Pressure Estimation Using Calibrated Cardiovascular Dynamics in the Photoplethysmogram	Proposing a DL-based framework that uses PPG signal for the cuffless continuous estimation of blood pressure
		Rabbani & Khan [22]	Contrastive Self-Supervised Learning for Stress Detection from ECG Data	Proposing a contrastive SSL model for stress assessment using ECG signals
		Pradhan et al. [23]	Classification of Autism and Control Gait in Children Using Multisegment Foot Kinematic Features	Analyzing MSF kinematics of ASD patients using multiple ML models to classify autism gait patterns
		Asfour et al. [24]	Feature–Classifier Pairing Compatibility for sEMG Signals in Hand Gesture Recognition under Joint Effects of Processing Procedures	Investigating the existence of feature–classifier pairing compatibility of sEMG signals in the context of Hand Gesture Recognition applications
		Abdeltawab et al. [25]	Predicting the Level of Respiratory Support in COVID-19 Patients Using Machine Learning	Showing that ML could be used to predict the required level of respiratory support for COVID-19 patients
Other	Computational models	Leong et al. [26]	A Surrogate Model Based on a Finite Element Model of Abdomen for Real-Time Visualisation of Tissue Stress during Physical Examination Training	Presenting an innovative surrogate model of abdomen mechanics by using ML and FE modeling to virtually render internal tissue deformation in real-time
		Padhee et al. [27]	Machine Learning for Aiding Blood Flow Velocity Estimation Based on Angiography	Proposing a framework to estimate hemodynamics in vessels based on angiography images using ML algorithms
	Bioinformatics	Thrun et al. [28]	A Bioinformatics View on Acute Myeloid Leukemia Surface Molecules by Combined Bayesian and ABC Analysis	Proposing a two-step approach (based also on Bayesian ML) to reconstruct the surface patterns on different subtypes of acute myeloid leukemia
		Bakare et al. [29]	Analytical Studies of Antimicrobial Peptides as Diagnostic Biomarkers for the Detection of Bacterial and Viral Pneumonia	Presenting data of AMPs to identify viral and bacterial pneumonia pathogens using in silico/ML technology
	Health management support	Ricciardi & Ponsiglione et al. [30]	Machine Learning and Regression Analysis to Model the Length of Hospital Stay in Patients with Femur Fracture	Generating several ML models that are capable of predicting the overall LOS following subjects’ femur fracture
		Trunfio et al. [31]	Implementation of Predictive Algorithms for the Study of the Endarterectomy LOS	Developing a forecasting model (also based on ML) of the LOS value following endarterectomy to investigate the main factors affecting LOS
